# Error in Study Type

**DOI:** 10.1001/jamahealthforum.2023.2428

**Published:** 2023-07-28

In the Original Investigation titled “One-Year Adverse Outcomes Among US Adults With Post–COVID-19 Condition vs Those Without COVID-19 in a Large Commercial Insurance Database,”^1^ published March 3, 2023, it was incorrectly stated in the Abstract, Key Points, Introduction, and Conclusions sections that this study was a case-control study. The correct study type is a cohort study. This article has been corrected.
